# Nanotechnology-Enabled Strategies for Enhancing Plant Abiotic Stress Tolerance

**DOI:** 10.3390/plants15203093

**Published:** 2026-10-10

**Authors:** Yusheng Li, Saddam Hussain, Lichen Wang, Siyu Chen, Wei Wang, Xiaoxiao Jing, Yaowen Wu, Jiahao Liu, Zaid Ulhassan, Mohammad Nauman Khan

**Affiliations:** 1 Laboratory of Plant Molecular Genetics & Crop Gene Editing, School of Life Sciences, Linyi University, Linyi 276000, China; hase705@nenu.edu.cn (S.H.); wanglichen@lyu.edu.cn (L.W.); 23329915.ok@163.com (S.C.); wangwei1226@lyu.edu.cn (W.W.); jingxiaoxiao86@163.com (X.J.); wuyaowen0728@163.com (Y.W.); 2 National Innovation Center of Saline-Alkali Tolerant Rice in Sanya, Sanya 572000, China; yushengli998@163.com; 3 School of Breeding and Multiplication, Sanya Institute of Breeding and Multiplication, Hainan University, Sanya 572000, China; zaidulhassan007@yahoo.com; 4 College of Agriculture, Tarim University, Alar 843300, China; 13163247758@163.com

**Keywords:** abiotic stress tolerance, nano-agrochemicals, reactive oxygen species (ROS) scavenging, phytohormone, antioxidant, nanozyme, multi-omics integrated analysis, environmental fate, risk assessment, sustainable agriculture

## Abstract

Amid increasingly complex climate scenarios, abiotic stresses such as drought, extreme temperatures, and soil salinization have emerged as primary bottlenecks limiting the sustainable development of modern agriculture. Because of their unique nanoscale effects and physicochemical properties, nanoparticles (NPs) can penetrate plant tissue barriers, offering a novel approach to enhance crop resilience to environmental stresses. This review systematically summarizes the core mechanisms by which NPs enhance plant stress tolerance. NPs primarily mitigate the overaccumulation of reactive oxygen species (ROS) and cellular oxidative stress by modulating antioxidant systems, thereby maintaining the physical integrity of photosynthetic apparatuses and cell membranes. Additionally, NPs regulate phytohormone signaling networks and the transcription of stress-responsive genes, while supporting ion compartmentalization and osmotic homeostasis in complex habitats such as coastal saline environments. Integrating multi-omics analyses, this review discusses plant metabolic responses to NPs and their dose-dependent toxicological effects. Addressing bottlenecks in practical application, we evaluate barriers to field-scale translation, the environmental release and migration of nanomaterials across water, air, and the food supply, and associated detection methods and safety risks, including the specific case of widely used silver nanoparticles. Future research must rely on multidisciplinary integration to optimize targeted delivery networks, premised on well-defined risk-assessment standards and viable cost–benefit balances, ultimately providing a scientific basis for enhancing agricultural climate resilience.

## 1. Introduction

Amidst intensifying global climate change, abiotic stresses have emerged as a primary environmental constraint on crop production. The global mean surface temperature has increased by approximately 1.1 °C above pre-industrial levels, and each 1 °C of warming is estimated to reduce the global yields of wheat by 6.0%, rice by 3.2%, maize by 7.4%, and soybean by 3.1% [1]. Drought frequency and severity have risen across many cropping regions, while altered precipitation patterns and prolonged meteorological droughts are projected to affect an even larger fraction of the global land surface. Concurrently, soil and water salinization is accelerating: the first global assessment of salt-affected soils in five decades estimates that approximately 1381 million hectares (10.7% of the global land area) are salt-affected, with roughly 10% of both irrigated and rainfed cropland impacted by salinization [2]. These stresses disrupt plant water homeostasis, inhibit photosynthesis, and induce cellular oxidative damage, severely constraining crop growth and yield [3]. Currently, conventional strategies to mitigate abiotic stress rely mainly on breeding stress-tolerant cultivars and applying traditional agrochemicals. However, conventional breeding is often hampered by long selection cycles and a limited pool of elite stress-tolerant germplasm [4]. Meanwhile, traditional agrochemicals generally have low field-use efficiency. A substantial proportion of active ingredients is readily lost via leaching, volatilization, or soil fixation [5], thus failing to match the dynamic physiological demands of crops under stress. Furthermore, prolonged and high-dose applications exacerbate agricultural pollution and drive soil degradation [6]. Therefore, there is a pressing need to develop highly efficient and eco-friendly technologies to optimize crop stress management.

In recent years, nanotechnology has emerged as a promising frontier for overcoming bottlenecks in enhancing plant stress tolerance [7]. Characterized by high specific surface areas, tunable surface chemistry, and nanoscale effects, nanoparticles (NPs) exhibit physicochemical properties fundamentally distinct from their bulk counterparts [8]. Their ultra-small size (typically 1–100 nm) enables them to effectively permeate cell wall pores and plasma membranes, bypassing the physical barriers that limit the uptake of conventional bulky agrochemicals—such as conventional synthetic fertilizers (e.g., granular urea), bulk metal-salt micro-nutrient fertilizers (e.g., ferrous sulfate and zinc sulfate), and traditional fungicide and herbicide formulations [9,10]. This unique size advantage facilitates trans-barrier transport and targeted release within plant tissues. Moreover, NPs with specific compositions possess intrinsic surface catalytic activities, enabling them to directly modulate ion homeostasis, reactive oxygen species (ROS) metabolism, and phytohormone networks, thereby triggering endogenous defense mechanisms [11,12]. In light of these advances, this review aims to systematically elucidate the core mechanisms by which nanotechnology enhances plant abiotic stress tolerance and to explore feasible pathways for field-scale applications. To this end, we first outline the physicochemical properties and translocation dynamics of NPs, then analyze their roles in reprogramming plant stress-response networks and their targeted applications across diverse abiotic stress scenarios. Concurrently, considering the barriers to translational research, we objectively assess the biosafety and environmental fate of nanomaterials, aiming to provide theoretical references for the targeted development of next-generation green agricultural inputs.

## 2. Literature Search Methodology

This review followed a structured and reproducible literature search. Peer-reviewed studies were retrieved from the Web of Science Core Collection, Scopus, PubMed, and Google Scholar, and were supplemented by authoritative institutional reports such as those of the Food and Agriculture Organization (FAO). Search strings combined the terms “nanoparticle”, “nanofertilizer”, or “nanotechnology” with “abiotic stress”, “drought”, “salinity”, “salt stress”, “heavy metal”, “cadmium”, “heat”, “chilling”, “temperature stress”, “antioxidant”, “nanozyme”, “phytohormone”, “omics”, “environmental fate”, and “risk assessment”. Priority was given to primary research published over the last decade, together with seminal and high-impact review articles. A study was included when it reported physiological, biochemical, molecular, or agronomic responses of higher plants to nanomaterials under a defined abiotic stress, and was excluded if it was confined to animal or microbial systems, lacked an identifiable plant response, was not peer reviewed, or was not in English. Titles and abstracts were screened against these criteria and the reference lists of key articles were hand-searched; the retained evidence was subsequently organized by stress type and mechanistic axis.

## 3. Nanoparticle Types and Plant Interactions

Nanotechnology is the design, characterization, production, and application of structures, devices, and systems by controlling shape and size at the nanoscale, conventionally defined as having at least one dimension between approximately 1 and 100 nm. At this scale, materials exhibit size-dependent optical, catalytic, magnetic, and mechanical properties that differ markedly from those of the corresponding bulk substances and dissolved ions [13]. In agriculture, nanotechnology is being developed for nanofertilizers and nanopesticides, controlled-release formulations, seed nanopriming, nanosensors for soil and crop monitoring, and remediation agents for contaminated soils and water [14].

### 3.1. Common Categories

Based on their core matrices, agricultural NPs are generally classified as metal(loid)-based, carbon-based, and biopolymer-based carriers [15]. Metal(loid)-based nanomaterials, predominantly represented by zinc oxide (ZnO), iron oxide (Fe_3_O_4_), and cerium oxide (CeO_2_) with mixed valence states, are the most extensively applied [16]. The dynamic redox transition between Ce^3+^ and Ce^4+^ endows these nanoparticles with exceptional, regenerative ROS-scavenging capabilities. These materials can serve as slow-release sources of trace elements in tissues or soils; released metal ions or intrinsic surface enzymatic activity can activate antioxidant systems and mitigate oxidative damage [17,18]. Silver nanoparticles (AgNPs), including widely used colloidal liquid formulations, are the predominant antimicrobial nanomaterials and are increasingly incorporated into agricultural biocides, coatings, and seed dressings through the sustained release of Ag^+^ ions, whose environmental mobility is a growing safety concern [19,20]. Carbon-based materials, such as carbon nanotubes (CNTs) and biomass-derived carbon dots, feature large specific surface areas and excellent electrical conductivity [21]. They can adsorb free nutrients in the soil, and once internalized by cells, facilitate electron transport within photosynthetic and respiratory chains [22,23]. To minimize active-ingredient loss, researchers often use biodegradable polymers (e.g., chitosan and polylactic acid) and porous inorganic carriers (e.g., mesoporous silica nanoparticles) as encapsulation matrices [24]. These materials enable the controlled release of active compounds through pore diffusion or stimuli-responsive degradation, offering superior biocompatibility and minimizing environmental accumulation. Table 1 summarizes the principal categories, representative materials, properties, and agricultural uses.

### 3.2. Uptake and Translocation Networks

The uptake and efficacy of NPs in plants are primarily dictated by their chemical composition, application modes, and physicochemical properties [14]. NPs predominantly enter plant systems through three primary delivery routes: foliar spraying, soil application, and seed nanopriming [25].

During foliar application, NPs smaller than 50 nm can directly enter stomata via air currents or permeate cuticular nanopores to reach epidermal cells [26], effectively overcoming the hydrophobic barrier of the leaf cuticle and potential interception by epidermal trichomes. Some NPs traverse membrane porins to enter organelles such as chloroplasts and participate in local metabolism [20]. This mode ensures rapid efficacy, directly delivering nutrients to photosynthetic organs while bypassing soil fixation [27].

Soil application provides a sustained nutrient supply, initiating with root internalization via sequential apoplastic and symplastic pathways [28]. Intercellular diffusion is ultimately restricted by the Casparian strip at the endodermis [16]. To breach this barrier, NPs must be internalized into the symplast via endocytosis or channel-mediated transport, enabling subsequent xylem-driven acropetal translocation to aerial tissues [29,30].

As a prophylactic strategy, seed nanopriming facilitates NP influx through seed coat micro-fissures and parenchymal interstitial spaces [31,32]. Following imbibition, internalized active components are rapidly reallocated to the developing radicle and plumule, minimizing nutrient competition with rhizosphere microflora [33]. Accordingly, seed-coat thickness, permeability, and the timing of imbibition set species-specific limits on nanopriming outcomes [14].

At the plasma membrane, internalization proceeds through aquaporins and ion channels, carrier-mediated transport, membrane porins, or energy-dependent endocytosis, with the dominant route depending on particle composition, size, and surface chemistry [10,29].

### 3.3. Influence of Physicochemical Properties on Translocation and Efficacy

**Figure 1:**
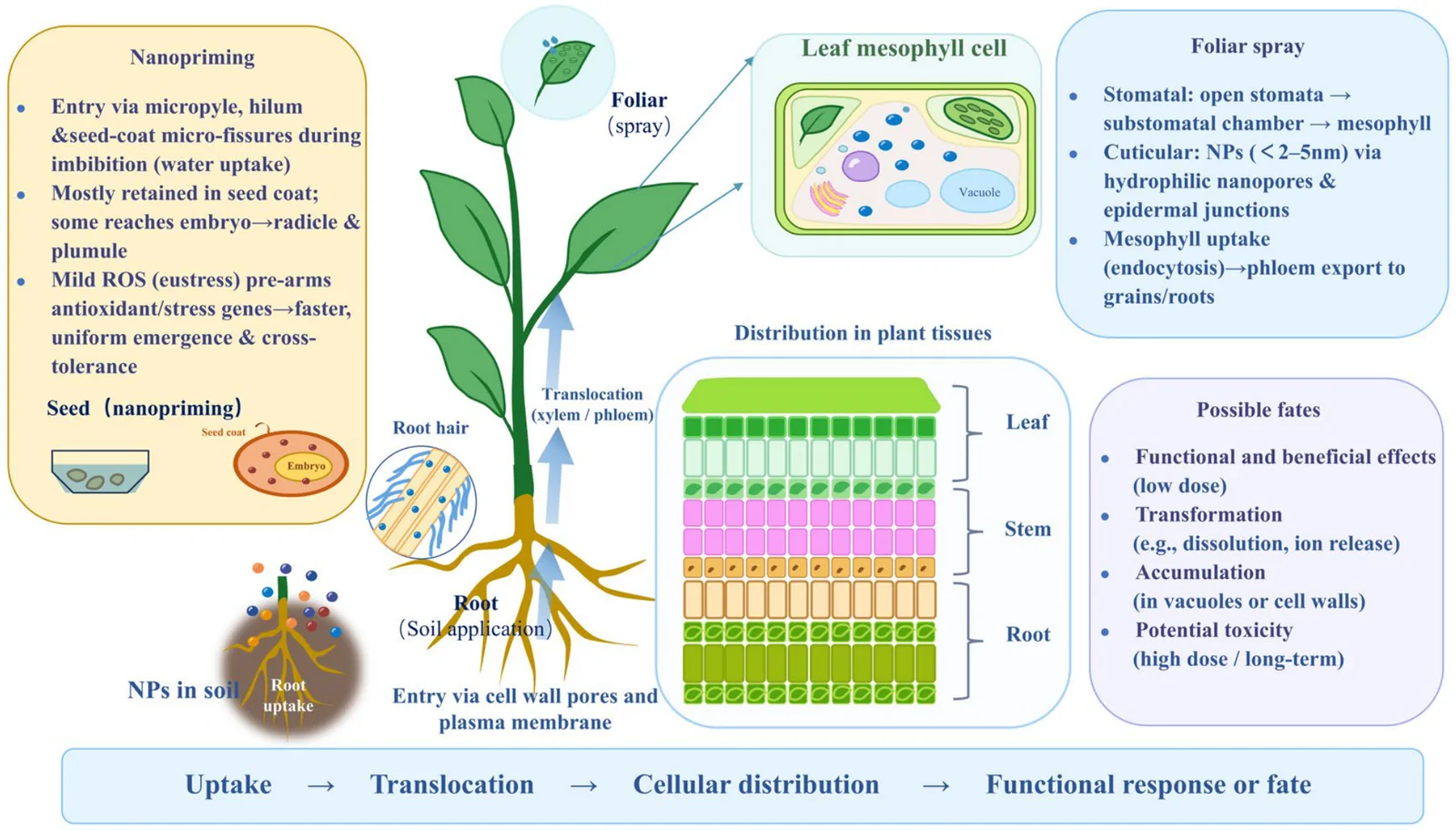
Uptake, translocation, and cellular fate of nanoparticles (NPs) in plants across the three delivery routes.

Particle size, surface charge, and surface modifications collectively govern the uptake, translocation efficiency, and bioavailability of NPs [16]. Particle size first sets access across tissue barriers: cell-wall pores generally admit particles below roughly 5–20 nm depending on species and tissue, whereas stomatal openings allow larger particles, commonly below 50 nm, to bypass the foliar cuticle [26,29]. Even after entry, ultra-small particles are readily lost through the transpiration stream, whereas overly large particles agglomerate on the root surface and remain in the cortex [16,34]. Surface charge then controls retention and mobility: the negatively charged pectic acids in cell walls electrostatically attract positively charged NPs and enhance uptake, whereas negatively charged NPs aggregate less with soil colloids and thus remain more mobile [35]. Surface functionalization governs dispersibility and ingress—hydrophilic, neutrally, or moderately charged coatings such as PEG, citrate, chitosan, and small biomolecules reduce agglomeration and facilitate passage through hydrated wall pores, whereas hydrophobic or strongly aggregating surfaces are retained at tissue surfaces; grafting such polymers or introducing ligands (e.g., EDTA) also protects active compounds and widens the safe dose window [16,26]. This fine-tuning aligns NP release with the physiological demands of plants under stress. Taken together, Figure 1 illustrates the complete journey of NPs, from root and foliar entry through xylem/phloem translocation and tissue distribution to subcellular localization and eventual fate.

## 4. Core Mechanisms of Stress Tolerance

**Figure 2:**
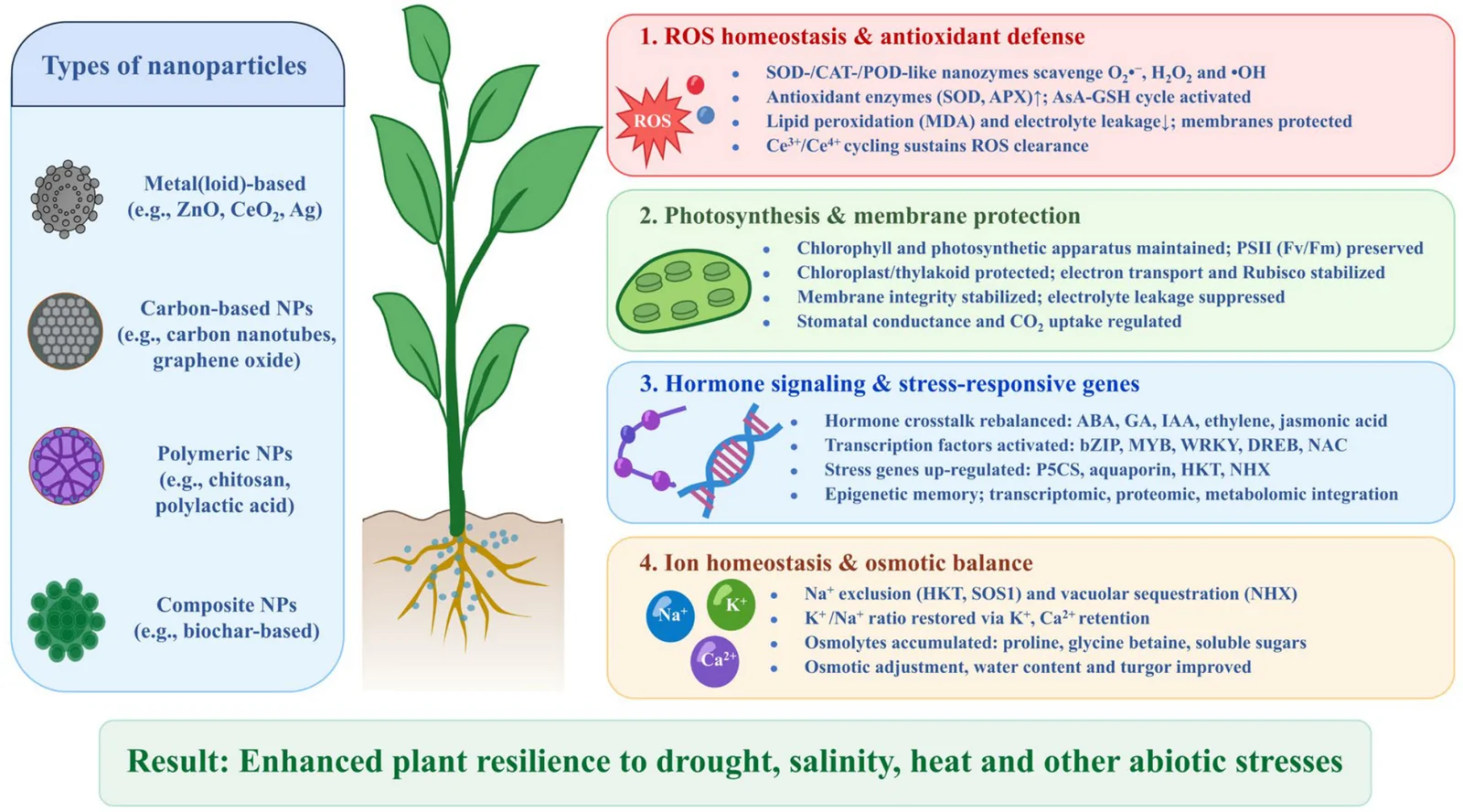
Integrated mechanisms by which nanoparticles enhance plant abiotic stress tolerance. Upward arrows (↑) indicate increase/upregulation, whereas downward arrows (↓) indicate de-crease/downregulation.

The efficacy of NPs in conferring abiotic stress tolerance stems from fundamental nano–bio interactions at the cellular and molecular levels. Diverging from conventional agrochemicals that merely act as exogenous elicitors, NPs operate through intrinsic catalytic activities, targeted subcellular localization, and profound molecular reprogramming to establish systemic plant resilience [36,37]. Figure 2 provides an integrated overview of the major NP classes, delivery routes, and four principal mechanistic axes: redox control, photosynthetic and membrane protection, hormonal and transcriptional reprogramming, and ion/osmotic homeostasis.

### 4.1. Intrinsic Nanozyme Activity and Redox Modulation

At the forefront of NP-mediated defense is the direct regulation of cellular redox homeostasis [38]. While conventional treatments indirectly upregulate plant antioxidant enzymes, certain nanomaterials (nanozymes) have intrinsic enzyme-mimicking properties that directly catalyze ROS degradation [39]. Beyond intrinsic surface catalysis, specific nano-formulations quantitatively amplify endogenous defense cascades. For instance, foliar application of calcium phosphate-urea nanocomposites (CaP-U NPs) to drought-stressed finger millet upregulated the activity and expression of key ROS-scavenging enzymes, elevating ascorbate peroxidase (APX) and superoxide dismutase (SOD) by 70% and 47.7%, respectively, relative to stressed controls [40]. Furthermore, nanomaterials like nanoceria, with their dynamic Ce^3+^/Ce^4+^ valence transitions, exhibit robust catalase- and superoxide dismutase-like activities. This enables them to neutralize free radicals directly at the site of generation [12,41]. Similarly, biomass-derived carbon dots utilize their high electron mobility and intrinsic catalase-like activity to scavenge ROS independently of the plant’s endogenous enzymatic capacity. This efficient redox modulation directly drives robust structural adaptations; for example, seed dressing with yellow-emissive carbon dots in cold-stressed oilseed rape increased the total root length and root tip number by 124.95% and 36.15%, respectively [42]. The mild, localized ROS generation induced by certain carbon nanomaterials can pre-activate the cellular antioxidant machinery without crossing the threshold of oxidative damage, effectively preparing the plant for subsequent severe environmental shocks [43,44].

### 4.2. Subcellular Targeting and Membrane Stability

The ultra-small dimensions of NPs enable them to bypass apoplastic barriers and achieve targeted sequestration within specific organelles [45]. High-resolution electron microscopy shows that nanoparticles can preferentially accumulate in critical sites such as chloroplasts and vacuoles [46]. Chloroplast localization allows NPs to directly stabilize the photosynthetic electron transport chain and modulate localized ion fluxes independent of plasma membrane transporters [47]. Moreover, as NPs permeate the cell wall, their interaction with apoplastic proteins and root exudates alters their surface properties, dictating their subsequent intracellular fate and interaction with cellular barriers [48]. Rather than functioning purely as abstract thermodynamic modulators, these materials practically influence membrane lipid composition and fluidity [49]. They help prevent cold-induced membrane rigidification or heat-induced hyper-fluidization, thereby maintaining crucial membrane integrity and preventing electrolyte leakage under environmental extremes [50,51]. Functionally, this structural preservation effectively mitigates stress-induced cellular damage. For instance, foliar application of cysteine-doped graphene quantum dots to drought-stressed plants suppressed lipid peroxidation (measured via MDA) and subsequent electrolyte leakage by 24.17% and 50.74%, respectively, securing intracellular homeostasis [52].

### 4.3. Transcriptional and Hormonal Reprogramming

The physiological acclimation induced by NPs is ultimately governed by extensive molecular reprogramming [53]. Transcriptomic analyses reveal that NP exposure acts as a rapid primary stimulus, activating broad-spectrum transcription factor networks (e.g., basic leucine zipper (bZIP), myeloblastosis (MYB)-family, and WRKY transcription fac-tors (WRKY proteins, named after the conserved WRKYGQK heptapeptide, Trp–Arg–Lys–Tyr–Gly–Gln–Lys, in their DNA-binding domain)) to prime defense-related genes [54]. Consistent with this transcriptional priming, the CaP-U NPs described in Section 5.1 were shown to induce changes in the expression of antioxidant- and defense-related genes in drought-stressed finger millet, rather than acting solely through surface catalysis [38]. This transcriptional reprogramming is measurable; for example, under severe salinity, applying silicon nanoparticles (SiNPs) to rice upregulates the critical sodium transporter gene *OsHKT1*;1 by 4-fold, directly supporting ionic and osmotic homeostasis [55]. These molecular events are systematically integrated via phytohormone signaling crosstalk [53]. The NP-induced spatial modulation of hormones, such as balancing growth-promoting indole-3-acetic acid (IAA) with stress-responsive abscisic acid (ABA), facilitates a critical metabolic shift [56]. This reallocates carbon and nitrogen resources from active proliferation toward survival-oriented structural fortification [57]. Beyond transient gene expression, emerging evidence suggests that NPs can induce specific DNA methylation patterns, conferring robust transgenerational stress memory in subsequent offspring [58].

### 4.4. Multi-Omics Insights

NP-mediated stress regulation involves intricate crosstalk among multiple metabolic pathways. Traditional single-tier molecular techniques fall short in fully elucidating this complexity, whereas multi-omics integrated analysis, encompassing transcriptomics, proteomics, metabolomics, and ionomics, offers a holistic systems biology perspective [59]. By coupling transcriptomics with metabolomics, researchers can trace NP-mediated alterations in metabolic networks with unprecedented resolution. Concurrently, proteomics and ionomics further decipher protein-level execution and elemental reallocation, respectively [60,61]. Transcriptomic data help identify differentially expressed genes, delineating their transcriptional responses in signal transduction and antioxidant defense [60]. Integrated with metabolomics, researchers can precisely quantify the differential abundance of key metabolites. Such combinatorial analyses unravel how NPs modulate carbon and nitrogen metabolic fluxes, providing empirical support for understanding energy reallocation and the biosynthesis of stress-responsive secondary metabolites under adversity [62].

## 5. Application to Specific Stresses

The efficacy of nanoparticles in abiotic stress management depends on the targeted modulation of physiological damage mechanisms induced by environmental adversity, as NPs reconfigure plant adaptive strategies across diverse stress-specific scenarios (Table 2, Table 3, Table 4 and Table 5).

### 5.1. Drought

Under drought conditions, prolonged water deficit induces severe cellular dehydration and curtails carbon assimilation. NP interventions mitigate these constraints by driving morphological adaptations, particularly root system optimization for deep-water foraging [63,64]. In staple crops like rice, applying manganese ferrite to the soil reshapes the root architecture by modulating localized IAA signaling, facilitating deeper soil penetration [57]. Beyond in planta structural modifications, engineered nanomaterials such as pectin-modified mesoporous silica nanoparticles directly remodel the rhizosphere [65]. These materials physically enhance soil moisture retention and alter local microbial communities, establishing a resilient hydraulic microenvironment that sustains root vigor during bulk soil desiccation [65,66].

Aboveground NP applications focus on minimizing transpirational water loss and maximizing water use efficiency (WUE) by physically fortifying cellular boundaries and regulating stomatal kinetics [66]. Cellulose nanocrystals specifically target cell wall metabolism in species like tall fescue, reinforcing the structural barrier against severe tissue dehydration [67]. Metallic NPs can upregulate drought-sensing peptides to stimulate systemic ABA biosynthesis [57,63]. This targeted signaling tightly couples’ stomatal closure with increased cuticular wax deposition, sealing the leaf surface to prevent visual senescence [57,68,69]. The field-scale agronomic utility of these nano-elicitors is governed by dose-dependent hormesis. While low concentrations of ZnO NPs optimize root volume and photosynthetic capacity, doses exceeding the physiological thresholds induce severe phytotoxicity and growth arrest [70]. To expand the therapeutic window and bypass the toxicity limits of single-element treatments, modern formulations increasingly use synergistic combinations. The co-application of SeNPs and CuNPs, for instance, safely maximizes plant water status by elevating leaf water content by up to 368.32% under severe drought relative to untreated controls, thereby broadening the threshold for field applications [47].

Key representative drought studies are summarized in Table 2. Proline- and betaine-doped ZnO NPs (15–21 nm), supplied to drought-stressed coriander, redirected secondary metabolism toward essential-oil production and promoted the accumulation of proline and betaine as seed osmoprotectants [71]. In arid-grown onion, ZnO supported on multi-walled carbon nanotubes acted as a smart micronutrient distributor, stimulating cell division (increasing the telophase index) and improving the spatial distribution of zinc during seed and seedling exposure [72]. For fenugreek, biosynthesized magnetite NPs (19–21 nm) applied as a foliar spray enhanced electron transport and broad-spectrum drought acclimation, improving photosynthetic performance and yield [73]. Green-synthesized AgNPs likewise enhanced the drought tolerance of cotton cultured in vitro, preserving relative water content and photosynthetic pigments while inducing antioxidant and drought-responsive genes [74]. Across these examples, smaller particles and foliar or seed-stage delivery tended to produce rapid benefits, whereas dose, coating, and crop-specific anatomy determined the magnitude of the response.

**Table 2:** Effects of nanoparticles on plant drought-tolerance mechanisms (↑, increase; ↓, decrease).

Stress Type	Plant Species	Nanomaterial & Properties	Application Mode & Dose	Core Mechanisms	References
Drought	Ginseng (*Panax ginseng*)	Pectin-modified mesoporous silica NPs; ~113.6 nm	Soil incorporation; 2 g/kg	Rhizosphere hydraulic microenvironment optimization (soil moisture retention ↑); osmotic adjustment (proline ↑); oxidative damage mitigation (MDA ↓)	[65]
Drought	Coriander (*Coriandrum sativum*)	Proline/betaine-doped ZnO NPs; ~15–21 nm	Soil supplementation; 50 mg/kg	Secondary metabolism redirection (essential oils ↑); seed osmoprotectant accumulation (proline, betaine ↑); oxidative stress alleviation	[71]
Drought	Peppermint (*Mentha piperita*)	Chitosan-coated Fe_3_O_4_ NPs & Kitoplus^®^; ~10–100 nm	Foliar spray; 5–10 μM + 0.5–1% Kitoplus	Stomatal kinetics regulation (conductance ↑); PSII photochemistry preservation (Fv/Fm ↑); essential oil biosynthesis ↑	[68]
Drought	Apricot (*Prunus armeniaca*)	Cysteine-doped graphene quantum dots; ~15 nm	Foliar spray; 10 mg/L	Leaf water status maintenance; enzymatic & non-enzymatic antioxidant cascades ↑; membrane integrity preservation (MDA, EL ↓)	[52]
Drought	Finger millet (*Eleusine coracana*)	Calcium phosphate-urea nanocomposite; ~15–50 nm	Foliar spray; 0.1% or 0.5%	Primary ROS scavenging enzymes ↑ (SOD, GPX, APX); osmoprotectant accumulation (proline ↑); photosynthetic pigments ↑	[40]
Drought	Cucumber (*Cucumis sativus*)	Multi-walled carbon nanotubes (<7 nm) & shungite powder	Soil incorporation; 0.067% (*w*/*w*)	Visual senescence delay (wilting ↓); transcriptomic upregulation of stress-responsive genes ↑; oxidative damage biomarkers ↓	[69]
Drought	Rice (*Oryza sativa*)	Manganese ferrite NPs (MnFe_2_O_4_ NPs); 20–60 nm	Soil application; 10 mg/kg	Root architecture remodeling (IAA signaling ↑); systemic ABA biosynthesis ↑ via drought-sensing peptide (CLE25 ↑); cuticular wax deposition ↑	[57]
Drought	Tomato (*Solanum lycopersicum*)	Functional carbon nanodots; 2–8 nm	Soil supplementation; 3 mg/kg	Water use efficiency (WUE) ↑; rhizosphere microbial community enrichment; primary osmolytes ↑ (proline, soluble sugar)	[66]
Drought	Apple (*Malus domestica*)	Graphene oxide; 2D nanomaterial	Foliar spray; 0.1–1 mg/L	Transcriptional activation of targeted drought-responsive factors (*MdDREB2A*, *MdERF1*↑); amino acid osmolyte ↑ (GABA, proline); deep rooting promotion	[64]
Drought	Onion (*Allium cepa*)	ZnO/MWCNT nanocomposite	Seed/seedling exposure; 15 μg/mL	Cell division stimulation (telophase index ↑); micronutrient (Zn) spatial distribution ↑	[72]
Drought	Soybean (*Glycine max*)	ZnO NPs; ~30 ± 10 nm	Foliar spray; 50–200 mg/L	Dose-dependent hormesis (low-dose: stomatal conductance & root volume ↑; high-dose > 200 mg/L: phytotoxicity ↑ & growth ↓)	[70]
Drought	Strawberry (*Fragaria* × *ananassa*)	Chitosan-stabilized Se NPs (~5–18 nm) & Cu NPs (~35–42 nm)	Foliar spray; 100 mg/L each	Synergistic dual-element efficacy; WUE & PSII performance ↑; antioxidant enzyme cascades ↑; lipid peroxidation ↓ (MDA ↓)	[47]
Drought	Tall fescue (*Festuca arundinacea*)	Si NPs (~20 nm) & Cellulose nanocrystals (CNCs; ~88 nm)	Foliar spray; 300 mg/L (Si NPs) or 100 mg/L (CNCs)	Material-specific targeted pathways (CNCs: cell wall synthesis/metabolism ↑; Si NPs: root nutrient uptake ↑); transcriptional reprogramming	[67]
Drought	Fenugreek (*Trigonella foenum-graecum*)	Biosynthesized magnetite NPs; 19–21 nm	Foliar spray; 50 mg/L	Electron transport rate ↑; broad-spectrum drought acclimation; photosynthetic performance & yield ↑	[73]
Drought	Cotton (*Gossypium hirsutum*)	Green-synthesized AgNPs (cotton seed oil cake extract); 50–100 nm; zeta −28.7 mV	In vitro culture; MS medium with PEG-induced drought; AgNP amendment	Relative water content, biomass & photosynthetic pigments preserved; osmolyte accumulation; antioxidant & drought-responsive genes upregulated (*CAT*, *POD*, *Cu*/*Zn SOD*, *MnSOD*, *MPK17*, *CAX2* ↑)	[74]

Abbreviations: NPs, nanoparticles; MWCNT, multi-walled carbon nanotube; ZnO, zinc oxide; Fe_3_O_4_, iron(II, III) oxide; MnFe_2_O_4_, manganese ferrite; Se, selenium; Cu, copper; Si, silicon; ROS, reactive oxygen species; SOD, superoxide dismutase; APX, ascorbate peroxidase; GPX, glutathione peroxidase; PSII, photosystem II; Fv/Fm, maximum photochemical efficiency of PSII; WUE, water use efficiency; MDA, malondialdehyde; EL, electrolyte leakage; IAA, indole-3-acetic acid; ABA, abscisic acid; GABA, γ-aminobutyric acid.

### 5.2. Salinity

Coastal saline environments impose a dual challenge on crops like rice: immediate osmotic shock followed by severe intracellular ion toxicity [75]. Nanoparticle interventions address this toxicity by modifying ion partitioning and compartmentalization [76]. At the systemic level, foliar applications of SiNPs in rice upregulate *HKT* transporter genes to drive active Na^+^ exclusion while preserving K^+^ and Ca^2+^ retention [55]. This efficacy is often linked to precise subcellular localization. Polyacrylic acid-coated nanoceria accumulate directly within chloroplasts to orchestrate *HKT1*-dependent ion partitioning, thereby securing a nearly 5-fold higher K^+^/Na^+^ ratio than in untreated stressed plants. This process operates independently of the traditional SOS1 or vacuolar sequestration mechanisms [77]. Organelle-level targeting simultaneously facilitates the remodeling of cell wall hemicellulose and pectin, preserving mesophyll conductance and carbon assimilation under extreme salinity [78]. The penetration of metal oxide NPs, such as Mn_2_O_3_, through the seed coat triggers the formation of a nanoparticle-protein corona [48]. This interaction directly modifies macromolecular profiles, such as lignin biosynthesis, to strengthen early physical barriers against salt ingress. Rather than merely scavenging existing free radicals, seed priming with carbonaceous materials like multi-walled carbon nanotubes induces a beneficial mild stress, or eustress [79]. This slight, transient H_2_O_2_ accumulation acts as a primary signal to pre-activate downstream enzymatic defenses long before severe oxidative damage begins [43].

The broader salinity literature, summarized in Table 3, reinforces these patterns across materials and delivery routes. Selenocystamine-modified chitosan nanomaterials applied as a root drench to rice and pakchoi activated SOD, CAT, and POD, enhanced MAPK-linked transcriptional reprogramming, enriched the rhizosphere microbial community, and reduced lipid peroxidation [80]. Tannic acid-iron nanomaterials delivered through seed priming or drenching acted as broad-spectrum ROS scavengers, lowering H_2_O_2_ and hydroxyl radicals while preserving membrane integrity and promoting biomass accumulation and root elongation in rice [81]. In wheat, licorice- and wolfberry-derived nanomaterials stimulated the AsA–GSH cycle and nitrogen-metabolism enzymes during seed priming, restoring the K^+^/Na^+^ ratio and preserving photosynthetic pigments [38]. Biologically synthesized magnesium oxide NPs primed radish seeds to increase carbohydrate and protein biosynthesis, suppress anti-nutritional tannins, and activate polyphenolic and flavonoid defenses [82]. Silicon nanoparticle priming of chia optimized the fatty acid profile, lowered the Na^+^/K^+^ ratio, and induced proline, CAT, and SOD responses [83], while foliar selenium NPs preserved photosynthetic pigments, activated CAT, APX, and polyphenol oxidase, and reduced lipid peroxidation in faba bean [84]. Foliar AgNPs similarly mitigated seawater-induced salt toxicity in chickpea, restoring shoot and root growth and fresh biomass, raising chlorophyll and Fv/Fm, and enhancing SOD and CAT activity [85]. Collectively, seed-priming and foliar routes dominate salinity management, and surface functionalization (e.g., chitosan, selenium, tannic acid) appears central to efficacy.

**Table 3:** Effects of nanoparticles on plant salinity-tolerance mechanisms (↑, increase; ↓, decrease).

Stress Type	Plant Species	Nanomaterial & Properties	Application Mode & Dose	Core Mechanisms	References
Salinity	Rice (*Oryza sativa*) & Pakchoi (*Brassica rapa* subsp. *chinensis*)	Selenocystamine-modified chitosan NMs; ~1 μm	Root drench; 300 mg/L	Antioxidant enzyme cascade activation (SOD, CAT, POD ↑); transcriptional reprogramming (MAPK signaling ↑); rhizosphere microbial community enrichment; lipid peroxidation alleviation (MDA ↓)	[80]
Salinity	Rice (*Oryza sativa*)	Tannic acid-iron nanomaterial; ~200 nm	Seed priming/drench; 25 μg/mL	Broad-spectrum ROS scavenging (H_2_O_2_, ·OH ↓); cell membrane integrity preservation; biomass accumulation & root elongation promotion	[81]
Salinity	Tomato (*Solanum lycopersicum*)	MWCNTs & multi-layer graphene	Seed priming; 50–500 mg/L	Mild ROS priming/eustress (slight H_2_O_2_, MDA ↑); enzymatic & non-enzymatic antioxidant defenses activation; nutraceutical profile optimization (lycopene, flavonoids, TSS ↑)	[43]
Salinity	Wheat (*Triticum aestivum*)	Licorice/wolfberry-derived complex NMs; ~42.2 nm	Seed priming; 200 mg/L (3 h)	AsA–GSH cycle & nitrogen metabolism enzymes activation; ion homeostasis restoration (K^+^/Na^+^ ratio ↑); photosynthetic pigment preservation	[86]
Salinity	Carrizo citrange (*Citrus sinensis* × *Poncirus trifoliata*)	Chitosan/alginate-encapsulated ceria nanocomposite; ~170 nm	Foliar spray; 15 mg/L	Root hydraulic conductance enhancement (water absorption ↑); osmolyte accumulation (proline ↑); non-enzymatic antioxidant pool expansion (polyphenols ↑)	[87]
Salinity	Rice (*Oryza sativa*)	Se NPs/MWCNTs hybrid; (SeNPs on tubular MWCNTs)	Foliar application; 160 μg/mL	Synergistic dual-material efficacy; agronomic yield components maximization (fertile spikelets, 100-grain weight ↑)	[79]
Salinity	Sorghum (*Sorghum bicolor*)	Gibberellic acid-grafted chitosan nanoparticles; ~50 nm	Foliar spray; 20 mg/L	Stress alleviation biomarker (proline, glycine betaine overaccumulation ↓); nutrient acquisition enhancement (macro/microelements ↑); genotype-specific yield response	[88]
Salinity	Cotton (*Gossypium hirsutum*)	PNC; ~7.9 nm	Foliar infiltration; 0.9 mM	Subcellular targeting (chloroplast co-localization); anatomical optimization (mesophyll conductance ↑); cell wall composition remodeling (hemicellulose/pectin ratio); ROS scavenging & ion homeostasis (ROS ↓, K^+^ ↑)	[78]
Salinity	Rice (*Oryza sativa*)	Si NPs; ~15–24 nm	Foliar spray; 20 mg/L	Genotype-wide ion homeostasis; transporter-mediated Na^+^ exclusion & K^+^/Ca^2+^ retention (HKT expression ↑)	[55]
Salinity	Arabidopsis (*Arabidopsis thaliana*)	PNC; ~8.9 nm	Parental leaf infiltration; 0.45 mM	Transgenerational epigenetic reprogramming (DNA methylation modification); root architecture optimization in nanomaterial-free offspring; ROS homeostasis (POD activity ↑)	[58]
Salinity	Radish (*Raphanus sativus*)	Biologically synthesized magnesium oxide nanoparticles	Seed priming; 10–150 μg/mL	Primary metabolite biosynthesis ↑ (carbohydrates, proteins); anti-nutritional factor suppression (tannins ↓); polyphenolic/flavonoid antioxidant defense activation	[82]
Salinity	Chia (*Salvia hispanica*)	Si NPs; <100 nm	Seed priming; 200 mg/L	Fatty acid profile optimization (α-linolenic, linoleic acids ↑); ion homeostasis (Na^+^/K^+^ ratio ↓); osmolyte & antioxidant cascades activation (proline, CAT, SOD ↑)	[83]
Salinity	Faba bean (*Vicia faba*)	Se NPs; 10–45 nm	Foliar spray; 5–10 mg/L	Photosynthetic pigment preservation; activation of antioxidant enzyme cascades (CAT, APX, PPO ↑); lipid peroxidation alleviation (MDA ↓)	[84]
Salinity	Pepper (*Capsicum annuum*)	Mn_2_O_3_ NPs; ~50 nm	Seed priming; 0.1–1 mg/L	Seed coat penetration & nanoparticle-protein corona formation; macromolecular reprogramming (lignin, protein profile modification)	[48]
Salinity	Cotton (*Gossypium hirsutum*)	PNC; ~6.1 nm	Foliar delivery; 0.9 mM	Subcellular targeting (chloroplast co-localization); non-canonical gene-level ion partitioning (*HKT1*-dependent Na^+^ exclusion, *SOS1*-independent); cytosolic K^+^/Na^+^ homeostasis restoration	[77]
Salinity	Chickpea (*Cicer arietinum*)	Biosynthesized AgNPs (Juniperus procera seed extract); spherical; ~100 nm	Foliar spray; 300–400 ppm (plants under 10–50% seawater)	Seawater salt toxicity alleviated; shoot/root growth & fresh biomass restored; chlorophyll, carotenoids, CSI & Fv/Fm ↑; MDA & TSS ↓; SOD & CAT activities ↑	[85]

Abbreviations: NMs, nanomaterials; NPs, nanoparticles; MWCNT, multi-walled carbon nanotube; Se, selenium; Si, silicon; Mn_2_O_3_, manganese (III) oxide; ROS, reactive oxygen species; SOD, superoxide dismutase; CAT, catalase; POD, peroxidase; APX, ascorbate peroxidase; PPO, poly-phenol oxidase; MAPK, mitogen-activated protein kinase; AsA-GSH, ascorbate-glutathione; TSS, total soluble solids; MDA, malondialdehyde; H_2_O_2_, hydrogen peroxide.

### 5.3. Heavy Metals

Heavy metal toxicity disrupts plant physiology primarily by substituting essential divalent cations in enzymes, inducing severe ROS bursts and impairing nutrient uptake. NPs alleviate this stress through two primary, sometimes opposing, strategies: immobilizing metals in the rhizosphere to enforce exclusion, or enhancing plant uptake to support phytoremediation [89,90]. For food crops, soil amendments with nanoscale zero-valent iron (nZVI) coupled with biochar represent a spatially targeted exclusion strategy. Biochar immobilizes cadmium via surface adsorption, while nZVI modifies root iron transporters; this synergy restricted cadmium partitioning into sensitive tissues [91]. In remediation-oriented species, such as water hyacinth, chitosan-grafted magnetic nanoparticles are paired with synthetic rhizobacteria. This biotic–abiotic association dramatically increases aqueous cadmium and lead capture while reprogramming the root microbiome to favor metal-tolerant Enterobacteriaceae [92].

While root interception limits initial exposure, suppressing the acropetal translocation of any internalized heavy metals is critical for protecting aerial tissues and the food supply. Foliar applications of zinc oxide nanoparticles in cotton, spatially decoupled from soil plant-growth-promoting rhizobacteria, repress the expression of cadmium transporters and modify cell wall polysaccharide metabolism. This blocks the re-distribution of cadmium into photosynthetic tissues [93]. Conversely, in dedicated hyperaccumulators, certain nanomaterials are deliberately deployed to enhance, rather than restrict, metal capture and translocation. Molybdenum trioxide nanoparticles stimulate cadmium phytoextraction in Solanum nigrum, with nontargeted metabolomics linking the response to carbohydrate and antioxidant remodeling and phytohormone signaling [94].

Table 4 summarizes representative heavy-metal studies spanning immobilization, exclusion, and enhanced phytoextraction. In soil-cultivated cucumber, a suite of met-al(loid) oxide and metal sulfide nanomaterials (e.g., MoS_2_; 5–120 nm) suppressed the bioaccumulation of As, Cd, Pb, Cr, Cu, and Ni, although antagonistic effects on the uptake of Mg, Fe, K, and Mn were also observed [95]. Green-synthesized Cu-Zn nanocomposites applied to Pb-stressed pea improved growth, isozyme activity, phenols, and photosynthetic traits, with remodeling of protein profiles [96]. A multi-omics follow-up in Solanum nigrum distinguished nanoparticle-specific regulation from that of Mo^6+^ ions and linked the response to heavy-metal-tolerant proteins and cadmium spatial reallocation [94]. Sugarcane pressmud-derived nano-biochar alleviated chromium stress in black cumin, lowering MDA and H_2_O_2_ while activating CAT, SOD, POD, and APX and preserving primary metabolism [97]. Graphene oxide enhanced root and shoot cadmium accumulation and improved carbon-sequestration and oxygen-release traits in the hyperaccumulator Lonicera japonica [98], whereas multi-walled carbon nanotubes restricted grain cadmium and lead below food-safety thresholds in scented rice while modulating the rhizosphere microbiome [99]. These studies highlight a crop-dependent duality: nanomaterials can either increase metal capture for remediation or enforce exclusion to protect the food supply.

**Table 4:** Effects of nanoparticles on plant responses to heavy-metal stress (↑, increase; ↓, decrease).

Stress Type	Plant Species	Nanomaterial & Properties	Application Mode & Dose	Core Mechanisms	References
Cd	White clover (*Trifolium repens*)	Nanoscale zero-valent iron combined with biochar; 40–100 nm	Soil amendment; nZVI (300 mg/kg) + BC (2.5%)	Synergistic heavy metal partitioning (soil Cd immobilization via BC; tissue Cd uptake via nZVI ↑); phytoremediation efficiency enhancement; photosynthetic pigment preservation	[91]
Multi-metal: Pb, As, Cd	Cucumber (*Cucumis sativus*)	Metal/metal-sulfide & oxide NMs (e.g., MoS_2_); 5–120 nm	Soil amendment; 100 mg/kg	Heavy metal bioaccumulation suppression (As, Cd, Pb, Cr, Cu, Ni ↓); antagonistic nutrient perturbation (Mg, Fe, K, Mn uptake ↓); NM constituent translocation (Mo, Si ↑)	[95]
Cd, Pb	Water hyacinth (*Eichhornia crassipes*)	Chitosan-grafted MNPs; 15–20 nm	Root immersion co-application; 100 mg/L MNPs + synthetic *E. coli*	Biotic–abiotic synergistic phytoremediation (aqueous Cd, Pb removal ↑); rhizosphere microbiome reprogramming (*Enterobacteriaceae enrichment*); tissue damage protection	[92]
Pb	Pea (*Pisum sativum*)	Green-synthesized Cu-Zn NCs; 25–70 nm	Foliar spray; 50–100 mg/L	Pb toxicity alleviation (growth indices ↑); antioxidant defense activation (isozymes, phenols ↑); macromolecular reprogramming (protein pattern remodeling)	[96]
Multi-metal: Cd, As, Zn	Black nightshade (*Solanum nigrum*)	MoO_3_ NPs; ~100 nm	Soil amendment; 4–40 mg/kg	Phytoremediation efficiency enhancement (Cd hyperaccumulation ↑); non-targeted metabolomic reprogramming (carbohydrates, antioxidants ↑); phytohormone signaling mediation	[100]
Cd	Cotton (*Gossypium hirsutum*)	ZnO NPs; ~18.4 nm	Spatially decoupled co-application: Foliar ZnO NPs + Soil PGPB	Spatially decoupled metal distribution modulation (Cd transporter repression, cell wall polysaccharide metabolism ↓); transcriptomic reprogramming (photosynthetic genes ↑)	[93]
Cd	Black nightshade (*Solanum nigrum*)	MoO_3_ NPs; ~100 nm	Hydroponic co-exposure; 5 μmol/L	Root-mediated multi-omics regulatory divergence; multi-dimensional transcriptomic-metabolomic reprogramming (heavy metal-tolerant proteins ↑); Cd spatial reallocation	[94]
Cr	Black cumin (*Nigella sativa*)	Sugarcane pressmud-derived nBC	Foliar spray; 100–150 mg/L	Intracellular oxidative stress alleviation (MDA, H_2_O_2_ ↓); antioxidant enzyme cascades activation (CAT, SOD, POD, APX ↑); primary metabolism & osmoprotectant preservation	[97]
Cd	Japanese honeysuckle (*Lonicera japonica*)	GO; Single-layer, 0.5–5 μm	Hydroponic co-exposure; 10–100 mg/L	Phytoremediation efficiency enhancement (root/shoot Cd accumulation ↑); ecological functional traits optimization (carbon sequestration, oxygen release ↑)	[98]
Multi-metal: Pb, Cd	Water hyacinth (*Eichhornia crassipes*)	FCNs & N-doped FCNs	Hydroponic co-exposure; 1–30 mg/L	Aqueous phytoremediation efficiency enhancement (Pb, Cd removal ↑); intracellular heavy metal immobilization; tolerance-related gene expression reprogramming	[66]
Cd	Pea (*Pisum sativum*)	SiO2 NPs; ~50 nm	Hydroponic co-exposure; 50 mg/L	Systemic Cd exclusion (tissue Cd accumulation ↓); root system architecture (RSA) optimization (lateral root proliferation ↑); transcriptomic reprogramming of C/N homeostasis	[101]
Cd & Drought	Rice (*Oryza sativa*)	Biosynthesized iron oxide NPs & chemical hydrogel NPs	Concurrent soil amendment; 25–100 mg/kg	Acropetal Cd translocation restriction; transcriptional repression of Cd transporters (*OsHMA2*, *OsHMA3*, *OsLCT1* ↓); dual-stress (Cd & drought) mitigation synergy	[102]
Cd	Flowering Chinese cabbage (*Brassica rapa var. parachinensis*)	Salvia miltiorrhiza-derived carbon dots	Foliar spray; 0.5–2.0 mg/L	Transcriptional cascade suppression (downregulation of *BrTCP9*); Cd uptake & ROS burst uncoupling (repression of *BrNramp1.2*, *BrRbohA* ↓); intracellular oxidative damage alleviation	[103]
Multi- metal: Cd, Pb	Scented rice (*Oryza sativa*)	MWCNTs; 1D tubular	Soil amendment; 100–300 mg/kg	Agronomic safety & heavy metal partitioning (grain Cd/Pb restricted below food safety thresholds < 0.2 mg/kg); rhizosphere microbiome modulation; oxidative stress mitigation (H_2_O_2_ ↓)	[99]

Abbreviations: NMs, nanomaterials; NPs, nanoparticles; MNPs, magnetic nanoparticles; MWCNT, multi-walled carbon nanotube; FCN, functional carbon nanodot; nZVI, nanoscale zero-valent iron; PGPB, plant-growth-promoting bacteria; ZnO, zinc oxide; MoO_3_, molybdenum trioxide; MoS_2_, molybdenum disulfide; SiO_2_, silica; Fe, iron; Mo, molybdenum; Si, silicon; Cu, copper; Zn, zinc; ROS, reactive oxygen species; SOD, superoxide dismutase; CAT, catalase; POD, peroxidase; APX, ascorbate peroxidase; MDA, malondialdehyde; H_2_O_2_, hydrogen peroxide; C/N, carbon/nitrogen.

### 5.4. Temperature

Extreme temperatures compromise cellular integrity primarily by altering mem-brane fluidity and destabilizing the photosynthetic apparatus. NPs mitigate chilling and heat injury by preserving organelle ultrastructure and pre-activating temperature-responsive transcriptional networks [24,104]. Under heat stress, zinc oxide nanoparticles localize within vacuoles and chloroplasts, preserving mitochondrial and chloroplast integrity; in alfalfa, this organelle-level protection rescued up to 90% of the biomass otherwise lost to high-temperature injury [24]. At the reproductive stage, where heat sensitivity is greatest, foliar zinc oxide applied at flowering in mungbean maintained gas exchange and rescued pod and seed set [46]. For chilling, nanocomposites such as chitosan-putrescine directly modulate membrane lipid thermodynamics, increasing elasticity to prevent cold-induced rigidification while expanding non-enzymatic redox pools such as glutathione and ascorbate [50]. Foliar nano-priming with zinc oxide in japonica rice activated cold-responsive transcription factors (e.g., bZIP, MYB, and WRKY) and promoted post-stress biomass recovery [105].

Additional temperature studies, compiled in Table 5, extend these findings. Gold nanoparticles used in wheat seed priming stimulated adaptogenic cold hardening, pre-served the photosynthetic apparatus, and activated systemic antioxidant defenses during low-temperature hardening [106]. Titanium dioxide nanoparticles applied to chickpea under cold stress induced temporal transcriptomic reprogramming of early defense and chromatin-architecture genes and maintained membrane integrity, with subcellular sequestration in vacuoles and chloroplasts mitigating electrolyte leakage and MDA accumulation [54,107]. In indica rice, comparative prophylactic foliar sprays of transition- and rare-earth metal oxide nanoparticles (ZnO, Fe_2_O_3_, TiO_2_, CeO_2_) produced genotype-specific recovery trajectories, alleviated stress-induced proline accumulation, and restored biomass [108]. Across chilling and heat studies, organelle-targeted localization and early transcriptional priming emerged as recurring determinants of protection.

**Table 5:** Effects of nanoparticles on plant temperature-stress tolerance (↑, increase; ↓, decrease).

Stress Type	Plant Species	Nanomaterial & Properties	Application Mode & Dose	Core Mechanisms	References
Chilling	Lettuce (*Lactuca sativa*)	Chitosan-putrescine nanocomposites (Ch-Put); ~100 nm	Foliar spray; 1.0–2.5 mM	Membrane lipid thermodynamic modulation (elasticity ↑, cold-induced rigidification ↓); non-enzymatic redox pool expansion (GSH, ascorbate ↑); antioxidant cascade activation	[50]
Chilling	Rice (*Oryza sativa* ssp. *japonica*)	ZnO NPs; ~30 nm	Foliar nano-priming; 25–100 mg/L	Transcriptional network activation of cold-responsive factors (*OsbZIP52*, *OsMYB4/30*, *OsWRKY76/94* ↑); antioxidative defense transcriptional cascades (*OsCu/ZnSOD* ↑); post-stress biomass recovery promotion	[105]
Chilling	Wheat (*Triticum aestivum*)	Au NPs; ~15.3 nm	Seed priming; 20 μg/mL (24 h)	Adaptogenic cold acclimation (hardening) stimulation; photosynthetic apparatus preservation; systemic antioxidant defense activation	[106]
Chilling	Overwintering oilseed rape (*Brassica napus*)	Yellow-emissive carbon dots; ~2.3 nm	Seed dressing	Intrinsic nanozyme activity (abiotic ROS direct catalysis); intracellular light supplementation (UV-to-yellow conversion); targeted root system hyper-proliferation (root length, tip number ↑)	[42]
Chilling	Chickpea (*Cicer arietinum*)	TiO_2_ NPs; 7–40 nm	Foliar spray; 5 mg/L	Temporal transcriptomic reprogramming (rapid early-phase defense & chromatin architecture genes ↑); membrane integrity preservation (ELI ↓); cold-induced oxidative stress alleviation	[54]
Chilling	Chickpea (*Cicer arietinum*)	TiO_2_ NPs; 7–40 nm	Application under 4 °C stress; 2–10 mg/L	Genotype-dependent nano-permeability; subcellular targeted sequestration (vacuoles, chloroplasts); redox homeostasis restoration; membrane damage mitigation (ELI, MDA ↓)	[107]
Chilling	Rice (*Oryza sativa* ssp. *indica*)	Comparative transition & rare-earth metal oxide NPs (ZnO, Fe_2_O_3_, TiO_2_, CeO_2_)	Foliar prophylactic spray; Optimal doses	Genotype-specific physiological plasticity (post-stress recovery trajectories ↑); stress-induced proline accumulation alleviation (cellular injury ↓); agronomic trait restoration (biomass ↑)	[108]
Heat	Alfalfa (*Medicago sativa*)	ZnO NPs; ~13.3 nm	Foliar spray; 30–90 mg/L	Subcellular targeted localization (vacuoles, chloroplasts); organelle ultrastructural preservation (chloroplast, mitochondrial integrity ↑); membrane lipid peroxidation alleviation (MDA ↓)	[24]
Heat	Mungbean (*Vigna radiata*)	ZnO NPs; ~20 nm	Foliar spray; 15–60 mg/L (at flowering stage)	Phenophase-specific protection (flowering stage); photosynthetic apparatus protection (gas exchange maintenance ↑); agronomic yield rescue (pods/seeds per plant ↑)	[46]
Heat & Drought	Wheat (*Triticum aestivum*)	Green-synthesized ZnO NPs	Combined biotic–abiotic application: PGPR + ZnO-NPs	Biotic–abiotic synergistic dual-stress mitigation; phytohormone crosstalk modulation (IAA, ABA ↑); AsA-GSH cycle & primary ROS scavengers’ activation (GR, DHAR, APX ↑); electrolyte leakage ↓	[109]

Note: Abbreviations: NPs, nanoparticles; PGPB, plant-growth-promoting bacteria; ZnO, zinc oxide; TiO_2_, titanium dioxide; Fe_2_O_3_, iron(III) oxide; CeO_2_, cerium dioxide; GSH, glutathione; ROS, reactive oxygen species; SOD, superoxide dismutase; APX, ascorbate peroxidase; GR, glutathione reductase; DHAR, dehydroascorbate reductase; MDA, malondialdehyde; ELI, electrolyte leakage; IAA, indole-3-acetic acid; ABA, abscisic acid; AsA-GSH, ascorbate-glutathione; UV, ultraviolet.

### 5.5. Dose-Dependent Effects and Hormesis

**Figure 3:**
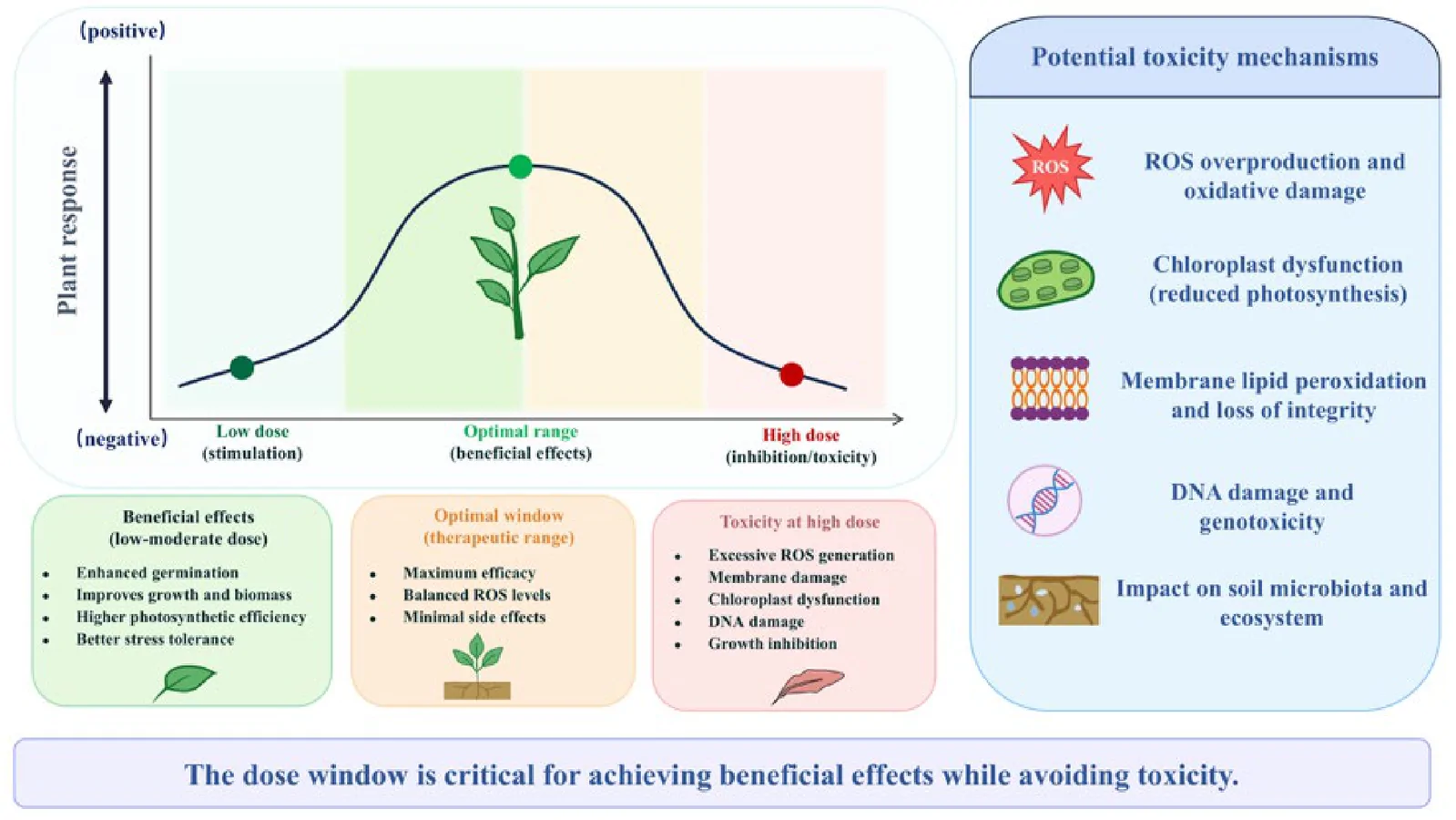
Dose-dependent hormetic effects and potential toxicity of nanoparticles in plants.

The biological effects of nanomaterials are intrinsically dose-dependent and typically exhibit a hormetic response characterized by low-dose stimulation and high-dose inhibition [110]. For instance, while foliar application of low-dose zinc oxide nanoparticles (≤200 mg/L) enhances photosynthetic efficiency by up to 49.1%, exceeding the critical threshold of 400 mg/L precipitates severe phytotoxicity, thereby suppressing this essential physiological trait by 66.3% and actively reducing overall plant biomass [70]. Beyond phenotypic manifestations such as reduced seed germination rates and inhibited root elongation, high-concentration NPs tend to agglomerate within tissues, hindering vascular transport and inducing cellular apoptosis [111]. Consequently, defining the safe dosage window and evaluating genotype-specific sensitivities are critical prerequisites for agricultural application. The high-dose inhibition shown in Figure 3 arises through several converging mechanisms: ROS overproduction and oxidative damage, chloroplast dysfunction and reduced photosynthesis, membrane lipid peroxidation and loss of integrity, DNA damage and genotoxicity, together with adverse effects on soil microbiota and the wider ecosystem.

## 6. Safety, Environmental Fate and Risk Assessment

Field-scale use of nanomaterials entails release into, and transfer across, multiple environmental compartments, calling for ecosystem-level rather than plant-centric risk assessment. In soil, pH, redox potential, and organic matter govern NP dissolution, transformation, and agglomeration, and accumulated particles can perturb rhizosphere microbial communities and the biogeochemical cycles they drive [112]; runoff, drainage, and wastewater carry NPs into surface water and groundwater, while spray drift, dust, and the aerosolization of dry formulations release them to the atmosphere, from which they are re-deposited onto land and water. Across these compartments, particles undergo aggregation, sulfidation, and dissolution and can affect algae, invertebrates, and fish, while residues that persist in edible tissues may be transferred through the food chain to higher trophic levels, with biomagnification still incompletely quantified [113]. Silver exemplifies this concern: the widely used colloidal AgNPs described in Section 3.1 slowly release Ag^+^ through oxidative dissolution, and the dissolved ion is generally more bioavailable and toxic than the particulate form to microbes, algae, plants, and invertebrates [19]. Such assessment depends on robust detection. Single-particle inductively coupled plasma mass spectrometry (spICP-MS) quantifies particle size distributions and number concentrations at environmentally relevant levels, even in complex waters, and is increasingly coupled with field-flow fractionation and ICP-time-of-flight for multi-element particles; electron microscopy (TEM/SEM), energy-dispersive X-ray spectroscopy, dynamic light scattering, nanoparticle tracking analysis, X-ray absorption spectroscopy, and emerging nanosensors provide the orthogonal characterization needed to distinguish engineered NPs from natural nanoparticles and background ions [114]. Table 6 maps the principal environmental compartments, their associated risks, and representative detection approaches [115,116].

## 7. Challenges and Future Directions

While the theoretical foundation for leveraging nanotechnology to enhance plant stress tolerance is robust, substantial barriers to practical translation persist. Current nano-agricultural research predominantly relies on controlled hydroponic or short-term pot systems. However, real-world agroecosystems feature fluctuating light and temperature, soil heterogeneity, and multiple interacting stresses. Therefore, transitioning to longitudinal field trials across diverse pedoclimatic zones is urgently required to systematically evaluate the long-term efficacy and physicochemical stability of nanomaterials in complex soil matrices [13].

The economic viability of nano-enabled cultivation must also be demonstrated. Although nanofertilizers and nanopriming can reduce application rates, nutrient losses, and management costs while raising yields and resource-use efficiency, these benefits must be weighed against high R&D and manufacturing expenses, quality-control and regulatory-compliance costs, specialized storage and infrastructure needs, and potential liabilities associated with environmental release [116]. Field-based cost–benefit and life-cycle analyses, across crop species and pedoclimatic zones, are needed to establish whether the agronomic gains of nanomaterial-treated cultivation reliably justify the increased input costs [114,116].

Developing next-generation nano-agrochemicals hinges on multidisciplinary integration to achieve smart, on-demand delivery. Formulating stimuli-responsive release systems is pivotal. Carriers engineered to respond to rhizospheric pH fluctuations, redox gradients, or specific enzyme exudations can seamlessly align release kinetics with dynamic physiological demands under stress [117,118]. Regarding application and targeting, integrating nanotechnology with precision agriculture and artificial intelligence algorithms enables the dynamic optimization of application parameters based on real-time monitoring data. Beyond technological innovation, establishing standardized ecotoxicological evaluation and regulatory frameworks for nano-agrochemicals is a fundamental prerequisite for their large-scale commercialization [119]. Because nanomaterials vary widely in physicochemical properties, traditional assessment frameworks struggle to gauge environmental exposure risks accurately. Developing quantitative evaluation standards to assess their environment fate systematically, impacts on non-target biological communities, and trophic transfer characteristics will provide a scientific, rigorous basis for the registration and standardized application of these novel agricultural inputs [120].

## 8. Conclusions

Nanoparticles have shown considerable potential to modulate plant responses to abiotic stresses. By facilitating ROS scavenging, preserving the structural integrity of cell membranes and photosynthetic apparatuses, and interacting with endogenous phytohormone and stress-responsive gene networks, nanomaterials offer intervention pathways distinct from traditional agrochemicals. Together, these mechanisms drive enhanced plant acclimation under a broad spectrum of adversities, including drought, salinity, heavy metal toxicity, and extreme temperatures.

Overcoming the translational bottleneck from promising laboratory-scale findings to practical field applications remains the primary challenge for nano-enabled agriculture. Future developments must strictly adhere to inherently safe design principles and undergo comprehensive evaluations across the entire product life cycle. Such assessments must encompass environmentally benign material synthesis, formulation stability, and long-term environmental degradation. Future research must integrate agronomy, materials science, and ecotoxicology to systematically optimize targeted delivery networks that mitigate ecological and food-web risks. Ultimately, this collaborative approach is essential for translating nanoscale interventions into reliable agronomic traits, thereby securing climate resilience and sustaining global agricultural productivity.

## Figures and Tables

**Table 1:** Common categories of nanomaterials used in plant science and agriculture.

Category	Representative Materials	Key Properties	Principal Agricultural Uses
Metal(loid)-based (metals and metal oxides)	ZnO, Fe_3_O_4_, CeO_2_, Mn_2_O_3_, TiO_2_, CuO, Ag, Au	High surface reactivity; catalytic (nanozyme) activity; some release micronutrient ions	Nanofertilizers, stress priming, antimicrobial agents, remediation
Carbon-based	Carbon nanotubes (SWCNT/MWCNT), graphene oxide, graphene quantum dots, carbon dots, nano-biochar	Large surface area; high conductivity and electron mobility; light conversion	Nutrient delivery, growth promotion, ROS modulation, remediation
Biopolymer-based	Chitosan, cellulose, polylactic acid, lignin, alginate	Biodegradable, biocompatible, stimuli-responsive	Encapsulation, controlled release, seed coatings
Porous inorganic/ceramic carriers	Mesoporous silica, mesoporous alumina, nanoclays	High pore volume, tunable pore size	Controlled-release carriers, rhizosphere amendment

Abbreviations: ZnO, zinc oxide; Fe_3_O_4_, iron(II,III) oxide; CeO_2_, cerium dioxide; Mn_2_O_3_, manganese(III) oxide; TiO_2_, titanium dioxide; CuO, copper(II) oxide; Ag, silver; Au, gold; SWCNT, single-walled carbon nanotube; MWCNT, multi-walled carbon nanotube; ROS, reactive oxygen species.

**Table 6:** Environmental compartments, principal risks of released nanomaterials, and representative detection and assessment approaches.

Compartment	Principal Exposure and Risk	Detection/Assessment Approaches
Soil	Altered microbial communities and biogeochemical cycles; transformation (dissolution, sulfidation); root uptake	spICP-MS, X-ray absorption spectroscopy, SEM/TEM, soil ecotoxicity assays
Surface water & groundwater	Aggregation/sedimentation; toxicity to algae, invertebrates, and fish; drinking-water exposure	spICP-MS, AF4-ICP-MS, nanoparticle tracking analysis, DLS
Air	Inhalation exposure from spray drift and dust; re-deposition onto land and water	Aerosol sampling, electron microscopy, mass spectrometry
Food/feed & food chain	Residues in edible tissues; trophic transfer and potential biomagnification	spICP-MS, ICP-MS (total), electron microscopy, speciation analysis

Abbreviations: spICP-MS, single-particle inductively coupled plasma mass spectrometry; AF4, asymmetric flow field-flow fractionation; ICP-MS, inductively coupled plasma mass spectrometry; DLS, dynamic light scattering; SEM/TEM, scanning/transmission electron microscopy; AgNPs, silver nanoparticles; NPs, nanoparticles.

## Data Availability

No new data were created or analyzed in this study.
